# Topical Amphotericin B Semisolid Dosage Form for Cutaneous Leishmaniasis: Physicochemical Characterization, Ex Vivo Skin Permeation and Biological Activity

**DOI:** 10.3390/pharmaceutics12020149

**Published:** 2020-02-12

**Authors:** Diana Berenguer, Maria Magdalena Alcover, Marcella Sessa, Lyda Halbaut, Carme Guillén, Antoni Boix-Montañés, Roser Fisa, Ana Cristina Calpena-Campmany, Cristina Riera, Lilian Sosa

**Affiliations:** 1 Department of Biology, Health and Environment, Laboratory of Parasitology, Faculty of Pharmacy and Food Sciences, University of Barcelona, 08028 Barcelona, Spainmcriera@ub.edu (C.R.); 2 Department of Pharmaceutical Technology and Physicochemistry, Faculty of Pharmacy and Food Sciences, University of Barcelona, 08028 Barcelona, Spain

**Keywords:** Amphotericin B, Sepigel 305^®^, *Leishmania infantum*, cutaneous leishmaniasis, topical treatment

## Abstract

Amphotericin B (AmB) is a potent antifungal successfully used intravenously to treat visceral leishmaniasis but depending on the *Leishmania* infecting species, it is not always recommended against cutaneous leishmaniasis (CL). To address the need for alternative topical treatments of CL, the aim of this study was to elaborate and characterize an AmB gel. The physicochemical properties, stability, rheology and in vivo tolerance were assayed. Release and permeation studies were performed on nylon membranes and human skin, respectively. Toxicity was evaluated in macrophage and keratinocyte cell lines, and the activity against promastigotes and intracellular amastigotes of *Leishmania infantum* was studied. The AmB gel remained stable for a period of two months, with optimal properties for topical use and no apparent toxic effect on the cell lines. High amounts of AmB were found in damaged and non-damaged skin (1230.10 ± 331.52 and 2484.57 ± 439.12 µg/g/cm^2^, respectively) and they were above the IC_50_ of AmB for amastigotes. Although there were no differences in the in vitro anti-leishmanial activity between the AmB solution and gel, the formulation resulted in a higher amount of AmB being retained in the skin, and is therefore a candidate for further studies of in vivo efficacy.

## 1. Introduction

Leishmaniasis are a group of diseases caused by species of *Leishmania* protozoan parasites and transmitted by the bite of *Phlebotomine* sand flies. Nearly 12 million people are infected in 98 countries worldwide [1]. There are three main forms of the disease and the manifestations depend on the infecting species, the immunological system of the patient and the area of inoculation [2,3]. Cutaneous leishmaniasis (CL), the most common form, can appear as single or multiple skin lesions, starting as a papule and potentially evolving into an ulcer; mucocutaneous leishmaniasis involves the destruction of mucous membranes; and visceral leishmaniasis (VL) is a fatal form affecting internal organs [4]. In the Mediterranean area, *Leishmania infantum* is the main causal agent of VL and CL [5], and particularly in Spain, *L. infantum* is the responsible for the endemic CL cases [6,7].

Although there is no universal consensus on the treatment of CL [8], pentavalent antimonials still remain the first-line therapy, frequently used in combination with pentoxifylline or paromomycin to maximize treatment efficacy despite the toxic side effects and the development of resistances [9]. Pentavalent antimonials (meglumine antimoniate and sodium stibogluconate) are administered intravenously or by painful intralesional injections which are frequently discontinued by patients, which promotes resistances [10].

Amphotericin B (AmB) is a polyene macrolide antibiotic that opens ion channels and aqueous pores in the *Leishmania* membrane by binding to ergosterol, making the bilayer thinner and increasing the permeability and loss of intracellular compounds, thereby triggering lysis and parasite death [11]. AmB intravenous injections are used as a second-line treatment for CL, but they cause severe toxicity, particularly nephrotoxocity in the case of conventional AmB. Liposomal AmB (Ambisome^®^) has been demonstrated to be more effective and less toxic, but its high cost makes its use difficult in developing countries [12,13].

In the last five years, research directed to improve delivery systems for the treatment of leishmaniasis has focused considerable attention on AmB as an active component [14], mainly using a parenteral administration approach. PLGA polymeric nanoparticles have been studied for intralesional and intravenous administration of AmB [4,15], as well as diverse emulsions and micelle formulations for intravenous use [16,17]. Considerable research is also being directed into the development of oral or topical formulations, as they imply greater patient compliance and fewer side effects. Chitosan has been investigated as a nanocarrier for oral administration [18], as well as an ingredient in an emulsion of nanoparticles [19]. Dermal delivery has been achieved with microneedles that generate micropores in the epidermis for the administration of an AmB solution [20]. For the topical treatment of CL, a combination of cyclodextrin plus AmB has been studied in a gel formulation [21], and ultradeformable liposomes have also been postulated [22].

Pharmaceutical gels are semisolid dosage forms consisting of a solvent thickened by the addition of colloidal substances. Colloids are polymers with gelling capacity and constitute a dispersed phase, and the liquid solvent is the continuous phase. The most common solvents used in gel formulation are water or hydroalcoholic solutions that form hydrogels. 

Hydrogels form cross-linked polymer chain networks able to capture large quantities of water in the spaces between chains and are widely used as vehicles in topical drug delivery [23]. They provide a refreshing sensation on the skin, are not sticky and have an optimal appearance. Sepigel 305^®^, which is composed of the fatty oil isoparaffin, the gelation-promoting polymer polyacrylamide and the non-ionic emulsifier laureth-7, forms hydrogels that permit the addition of hydrophilic and lipophilic substances.

The objective of this work was to develop a topical formulation incorporating AmB for CL therapy. AmB is a very hydrophobic molecule and is poorly soluble in water and most organic solvents. Using the capacity of Sepigel 305^®^ to include hydrophobic substances, we developed an AmB hydrogel-based formulation with optimal organoleptic characteristics. The stability was assessed by examining the physicochemical properties of the gel for two months. Permeation and retention studies were performed ex vivo in human skin, tolerance was checked in healthy volunteers, and toxicity and leishmanicidal activity were examined in vitro in promastigotes and intracellular amastigotes of *Leishmania infantum*.

## 2. Materials and Methods 

### 2.1. Reagents

Amphotericin B and Sepigel 305^®^ were purchased from Acofarma (Barcelona, Spain). Dimethyl sulfoxide (DMSO) and NaOH were obtained from Sigma-Aldrich (Darmstadt, Germany). TranscutolP^®^ was provided by Gattefossé (Barcelona, Spain). The distilled water used to perform the assays was acquired from a Mili-Q^®^ Plus System (Millipore Co., Burlington, MA, USA).

### 2.2. Parasite Strains and Cultures

For the parasitology experiments, the *Leishmania infantum* strain (MHOM/ES/2016/CATB101) was isolated from a patient with CL in Spain, and promastigotes were maintained at 26 °C in supplemented Schneider complete medium at pH 7.0 with 20% heat-inactivated fetal calf serum, 25 µg/mL gentamicin solution (Sigma, St. Louis, MO, USA), and 1% penicillin (100 U/mL)–streptomycin (100 mg/mL) solution (Sigma, St. Louis, MO, USA).

### 2.3. Gel Preparation

Briefly, the gel-based 1% Sepigel 305^®^ formulation was prepared with constant mechanical stirring (solution A). Subsequently, 150 mg of AmB was dissolved in 5 mL of DMSO, of which 1 mL was slowly added under agitation to 30 mL of solution A. The resulting solution was adjusted to pH 6 with NaOH 2N solution, thus obtaining AmB gel at a final concentration of 1000 µg/mL.

### 2.4. Physicochemical Characterization of the AmB gel

#### 2.4.1. Morphological Analysis

The internal morphological structure was observed under scanning electron microscopy (SEM) using a FEI Quanta 200 (Hillsboro, OR, USA) in high-vacuum conditions. AmB gel was dried off for 14 days under a vacuum desiccator and a sample of 0.1 g was covered with carbon.

#### 2.4.2. Swelling and Degradation Tests

Dried and fresh AmB gel were utilized to perform the swelling and degradation tests. In both studies, the AmB gel was kept in PBS (pH = 5.5) for 21 min at 32 °C. Samples (n = 5) were weighed after wiping the superficial water at determined timeslots every 3 min. The swelling ratio (SR) was calculated using the following equation:

$$\mathrm{SR}=\frac{Ws-Wd}{Wd}$$

where *Ws* is the weight of the swollen AmB gel at 3 min intervals and *Wd* is the weight of dried gel.

The degradation was expressed as the weight loss percentage (WL) and was calculated following the equation:

$$\mathrm{WL}\;\left(\%\right)\;=\;\frac{Wi\;-\;Wd}{Wi}\;\times \;100$$

where *Wi* is the weight of the AmB gel at the initial point of the experiment and *Wd* is the gel weight every 3 min at preselected time points.

#### 2.4.3. Porosity Study

The porosity percentage is based on the sinking of the AmB gel previously dried in absolute ethanol for 20 min and weighing it every 2 min when the excess of ethanol is blotted. 

The porosity percentage (P) was calculated with the equation:

$$P\;\left(\%\right)\;=\;\frac{W2\;-\;W1}{\rho \times V}\;\times \;100$$

where *W*1 stands for the weight of the dried AmB gel, *W*2 is the weight of the AmB gel after each immersion every 2 min, ρ is the density of ethanol and *V* is the volume of the AmB gel.

### 2.5. Stability Studies

Samples of the AmB gel were stored at different temperatures (room temperature (RT), 4 °C and 37 °C) to determine the stability of the formulation.

The pH values were registered with universal test paper (Ahlstrom-Munksjö, Helsinki, Finland) by directly spreading the samples (n = 3) and checking the visual appearance and drug loading after 1, 30 and 60 days.

The AmB content was analyzed using a previously validated High-Performance Liquid Chromatography (HPLC) methodaccording to international guidelines, with linearity, sensitivity, accuracy, precision and selectivity as described by Sosa et al. (2017) [24]. The calibration curves were prepared with freshly prepared stock solutions of AmB ranging from 0.39 to 200 µg/mL. The analytical method was precise, with coefficient of variation between 0.02% and 8.79%, a relative percent error between −1.16% and 3.46%, and linear within the range of concentrations used (0.39–200 µg/mL), with a p value corresponding to the ANOVA applied to the mean values of 0.05. AmB was determined using a Waters^®^ 515 HPLC Pump, a 717 Plus Autosampler and a 2487 Dual λ Absorbance Detector (Waters^®^, Milford, MA, USA). The assay was carried out using a Kromasil^®^ Eternity C18 (250 mm × 4.6 mm × 5 µm, Teknokroma, Barcelona, Spain). The mobile phase was a mixture of acetonitrile:acetic acid:water (52:4.3:43.7 *v*/*v*/*v*) and was pumped through the C18 column at a flow rate of 0.5 mL/min. A volume of 10 µL was injected per sample and finally the elute was analyzed at 406 nm. All measurements were performed at RT and at isocratic conditions of elution.

Destabilization phenomena were inspected by multiple light scattering and analysis of backscattering and transmission profiles using a TurbiScanLab^®^ (Formulaction Co., L’Union, France) with a pulsed near-infrared light source (γ = 880 nm) at 25 °C, 1, 30 and 60 days after the preparation of the AmB gel.

### 2.6. Rheological Studies

A rotational rheometer (Thermo Scientific HaakeRheostress 1, Thermo Fischer Scientific, Karlsruhe, Germany) with a cone plate set-up, a fixed lower plate and a mobile upper cone Haake C60/2° Ti was used to carry out the rheological assay. Measurements were taken in duplicate at 25 °C and 4 °C 24 h after gel preparation, as the rheometer is equipped with a ThermoHaake Phoenix II + Haake C25P (Thermo Fischer Scientific, Waltham, MA, USA) to control the temperature. The shear rate ramp program encompassed a ramp-up period from 0 to 50 s^−1^ for 3 min, a constant shear rate period of 50 s^−1^ for 1 min and a ramp-down period from 50 to 0 s^−1^ for 3 min. The data acquired from flow curves (τ = f($\dot{\gamma }$) were fitted to different mathematical models (Newton, Bingham, Ostwald-de-Waele, Herschel-Bulkley, Casson and Cross) and the best fit was selected according to the correlation coefficient value (r). Mean viscosity curve (η = f($\dot{\gamma }$)) values were determined from the constant share section at 50 s^−1^ [25].

### 2.7. Spreadability Test

The spreadability (n = 3) of AmB gel was determined by placing 0.5 g of the formulation between a plate and a millimeter glass plate cover. Different weights (2, 5, 10, 20, 50, 100, 200 and 300 g) were successively placed on top for 2 min each at RT. Spread diameters were measured and fitted into mathematical models using GraphPad Prism^®^ version 6.0 (GraphPad Software Inc., San Diego, CA, USA).

### 2.8. In Vitro Release Studies

AmB release was assessed in an 0.64 cm^2^ area of Franz diffusion cells (FDC 400; Crown Glass, Somerville, NJ, USA) on nylon membranes (0.45 µm) (Sterlitech Corporation, Kent, WA, USA). Pure DMSO solution continuously stirred at 600 rpm and 32 ± 0.5 °C was used as the receptor medium to accomplish sink conditions. Then, 0.75 g of AmB gel and 0.75 mL of AmB solution were deposited in the donor compartment. The study was performed in three replicates for a period of 48 h and the AmB was determined by HPLC-UV at preselected time points.

### 2.9. Ex Vivo Permeation Studies

Human skin from a donor undergoing plastic surgery at Barcelona-SCIAS Hospital (experimental protocol reference number: BEC/001/16, Barcelona, Spain) was used to perform the permeation assays in Franz diffusion cells, as described in the previous section. Skin was sliced into 400 μm thick specimens and the integrity was checked by measuring the transepidermal water loss (TEWL) parameters [26]. To damage the tissue, the stratum corneum of healthy skin was partially removed 7 times with the tape stripping technique. Then, both healthy and damaged skin were used in the experiments. After this, 0.75 g AmB gel was deposited on the upper side of the skin, facing the stratum corneum. Transcutol P^®^ was used as the receptor medium and was kept at 32 ± 0.5 °C in the receptor compartment. After 24 h, the amount of AmB in the receptor compartment as well as inside the skin was quantified by HPLC in three replicates. The drug retained in the skin was extracted after washing and cutting the permeation area with an ultrasound bath and cooling for 15 min.

### 2.10. In Vivo Tolerance Studies

Ten volunteers with healthy skin took part in the study, which was approved by the Ethics Committee of the University of Barcelona (reference number: IRB00003099; date: 20 March 2018), following the Declaration of Helsinki guidelines [27]. Participants were asked not to use any kind of cosmetics on the flexor side of the left forearm in the days before the test. Measurements were carried out at baseline, 30 min after the volunteers arrived in the test room, and then at 15 min, 1 h and 2 h after the application of 0.5 g of AmB gel. TEWL and the stratum corneum hydration (SCH) values were measured by a Tewameter^®^ TM 300 (Courage-Khazaka electronic GmbH) and a Corneometer^®^ CM 825 (Courage-Khazaka electronic GmbH), respectively, in accordance with international guidelines [28].

### 2.11. In Vitro Cytotoxicity Assay

Three different cell lines were used to establish the cytotoxic effect of the AmB gel—two macrophage cell lines (RAW 264.7 and J774A.1) and a keratinocyte cell line (HaCat). A suspension of 5 × 10^4^ cells/mL of each cell line was seeded in 96-well plates (Costar 3596, Corning Incorporated, NY, USA) and cultured at 37 °C and 5% CO_2_ atmosphere in RPMI-1640 complete medium [10% heat-inactivated fetal calf serum and 1% penicillin (100 U/mL)–streptomycin (100 mg/mL)] for 24 h. After that, serial dilutions of the AmB gel and AmB solution were added and the conditions were maintained for another 24 h. Finally, the reagent WST-1 (Roche Diagnostics GmbH) was added at a concentration of 10% to all wells and the plate was incubated for 4 h in the same conditions regarding temperature and CO_2_ atmosphere. Absorbances were read at 450 nm (Multiskan EX, Thermo Electron Corporation, Shanghai, China). To determine the concentration that inhibits 50% of cell viability (CC_50_), a linear regression analysis was performed, and the experiments were performed in triplicate [29].

### 2.12. In Vitro Leishmanicidal Activity against Promastigotes

Promastigotes were cultured in cell culture flasks. To assess their activity, serial dilutions of AmB gel and AmB solution were prepared in Schneider medium and placed in a 96-well plate (Costar 3596, Corning Incorporated, NY, USA). A suspension of 10^6^ promastigotes/mL in a logarithmic phase were added to the previous dilutions and incubated at 26 °C for 48 h. Parasitic growth was evaluated using the phosphatase acid method described by Carrió et al. (2000). Briefly, the samples were lysed, and the enzymatic reaction was revealed with *p*-nitrophenyl phosphate by alkalization. The optical density was read at 405 nm (Multiskan EX, Thermo Electron Corporation, Shanghai, China). The IC_50_ (the concentration that inhibits 50% of parasite growth) was calculated by variable transformation and linear regression analysis, and the experiments were performed in triplicate [30].

### 2.13. In Vitro Leishmanicidal Activity against Amastigotes

The cell line J774A.1 was used to study the activity of the AmB gel against amastigotes. A concentration of 5 × 10^4^ cells/mL was seeded in an 8 LabTek chamber slide system (Nunc^®^, Rochester, NY, USA) and incubated for 24 h at 37 °C in a 5% CO_2_ atmosphere. Late stationary phase promastigotes from a 5-day growth culture were added in a ratio of 1:10 (macrophage:parasites) and incubated for 24 h in the same conditions. After the elimination of free promastigotes by washing, fresh RPMI-1640 complete medium with serial dilutions of gel or solution was added, and incubation was carried out for 48 h in the same conditions. Untreated cultures were included as a positive control. Slides were fixed and stained with Giemsa to evaluate the number of amastigotes in 300 macrophages and the percentage of infected cells was determined in triplicate. The IC_50_ was expressed as the percentage of growth inhibition with respect to the untreated controls [31].

### 2.14. Statistical Analysis

The data collected in the studies were analyzed by one-way parametric analysis of variance (ANOVA), followed by a multiple comparison Tukey test (*p* < 0.05 = statistically significant differences) using Prism^®^ V. 5 (GraphPad Software Inc., CA).

## 3. Results

### 3.1. Physicochemical Characterization of the AmB gel

**Figure 1:**
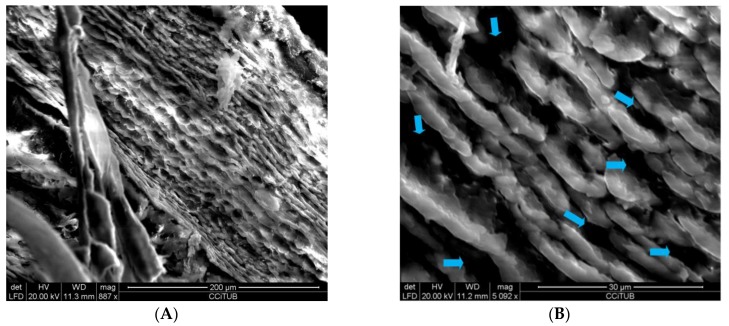
Images of amphotericin B (AmB) gel under SEM, (**A**) undulating layers (887×) and (**B**) detail of cavities between layers (5092×).

Figure 1 shows the homogeneous laminar disposition of undulating layers of AmB gel, with holes or cavities between the layers.

**Figure 2:**
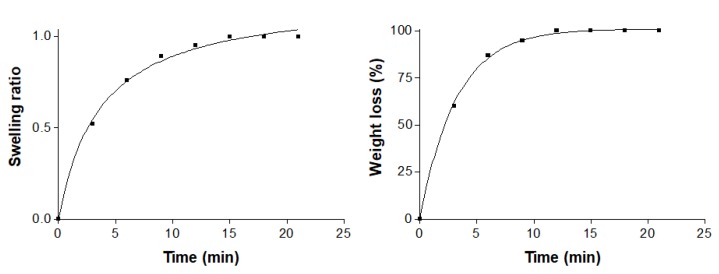
Swelling ratio (left) and degradation (right) of AmB gel during 21 min in PBS medium.

The AmB gel described a hyperbolic model in the swelling experiment, with a kinetic constant of k = 3.66 min (r^2^ = 0.9986). Gel degradation was completed in 10 min and was represented by a one-phase exponential model, where the kinetic constant was k = 0.31 min^−1^ (r^2^ = 0.9992) and the porosity percentage was ~85.47% ± 0.73%. The swelling and degradation graphs generated by the AmB gel are shown in Figure 2.

### 3.2. Stability Studies

The AmB gel presented a pH value of 5–6 throughout the experiment. The yellow colour of the AmB formulation remained unchanged at all temperatures tested in the study period. No lumps or precipitation were observed.

**Figure 3:**
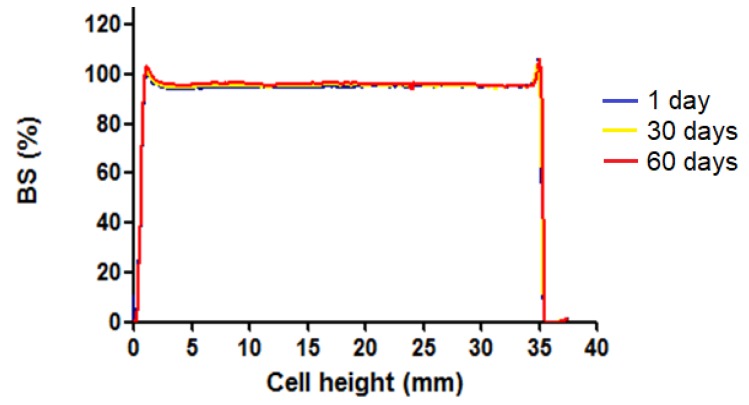
Laser backscattering (BS) profile of AmB gel over 60 days at 25 °C. The left side corresponds to the bottom of the vial containing the sample, whereas the right side corresponds to the sample behavior at the top.

Figure 3 shows the backscattering profile of Amb gel at 25 °C over a period from 1 to 60 days. No sedimentation, flocculation or coalescence phenomena were detected.

The initial AmB content revealed the gel formulation had 95.99% entrapment efficiency. The concentration of AmB in the gel over time is summarized in Table 1. The AmB content of the gel samples did not present statistical differences (*p* > 0.05) at the tested temperatures after 60 days of storage at 4 °C, whereas a significant decrease in the AmB content of the gel was observed when it was maintained at RT and 37 °C.

### 3.3. Rheological Studies

**Figure 4:**
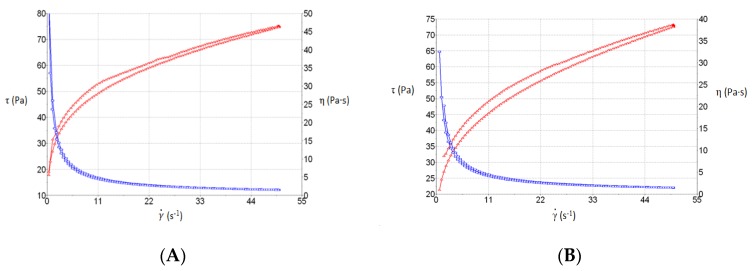
Rheological behavior of AmB gel showing a hysteresis loop. (**A**) Behavior at 25 °C and (**B**) 4 °C.

The AmB gel presented rheo-thinning (pseudoplastic) behavior at 25 °C and 4 °C (Cross model; r^2^ = 0.9996 and r^2^ = 0.9998, for the two temperatures, respectively). The viscosity at 50 s^−1^ was 1542.5 ± 3.49 mPa·s, and there was ahysteresis loop with an area of 194.4 Pa/s at 25 °C. The viscosity at 50 s^−1^ was 1457 ± 4.04 mPa·s, and there was also a hysteresis loop with an area of 160.1 Pa/s at 4 °C. Figure 4 shows the rheological graphs.

### 3.4. Spreadability Test

**Figure 5:**
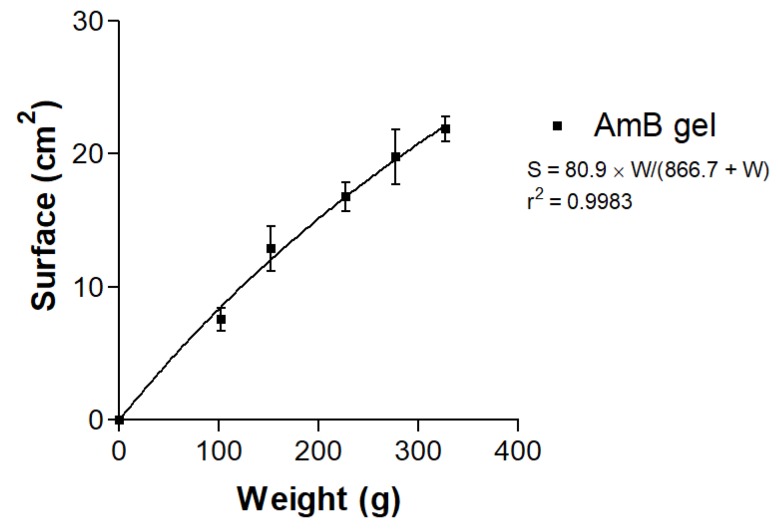
Graphicalhyperbolic behavior of AmB gel spreadability.

The spreadability of AmB gel followed a hyperbola kinetic model:

[S = S_max_ × W/(K_d_ + W)]

where S represents the extended surface in cm^2^, S_max_ is the maximum extension surface in cm^2^, W stands for the weight attached in g and K_d_ is the kinetic constant in g. Figure 5 shows the resulting graph and equation.

### 3.5. In Vitro Release Studies

**Figure 6:**
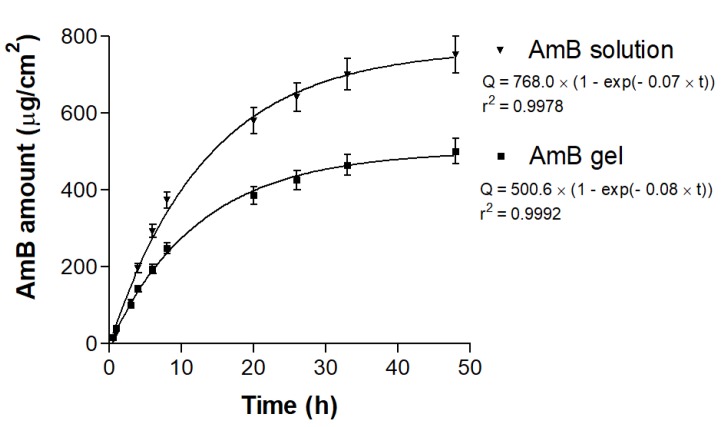
Released amounts (Q) of AmB from the solution and gel through nylon membranes for 48 h.

The amount of AmB released from the solution and gel at 48 h of the experiment was 753.01 ± 83.43 µg/cm^2^ and 501.02 ± 58.17 µg/cm^2^, respectively (Figure 6), which represents an AmB release of 100.40 ± 11.13%from the solution and 66.80 ± 8.9% from the gel. The mathematical model with the best fit was one-phase exponential association.

### 3.6. Ex Vivo Permeation Studies

Table 2 summarizes the amount of AmB obtained in the permeation studies with damaged and non-damaged skin. No AmB flux was observed across the skin barrier.

### 3.7. In Vivo Tolerance Studies

**Figure 7:**
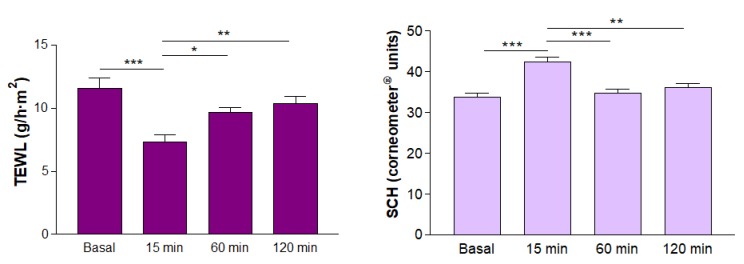
Biomechanical parameters: TEWL (transepidermal water loss) and SCH (stratum corneum hydration) values for 2 h after AmB gel application (* *p* < 0.05; ** *p* < 0.01; *** *p* < 0.001).

At 15 min of AmB gel application, decreased values of TEWL were observed, as well as an increased hydration of the stratum corneum, with statistically significant differences compared with all the time points in the experiment. The graph in Figure 7 shows the variations of biomechanical properties. 

### 3.8. In Vitro Cytotoxicity Assay

**Figure 8:**
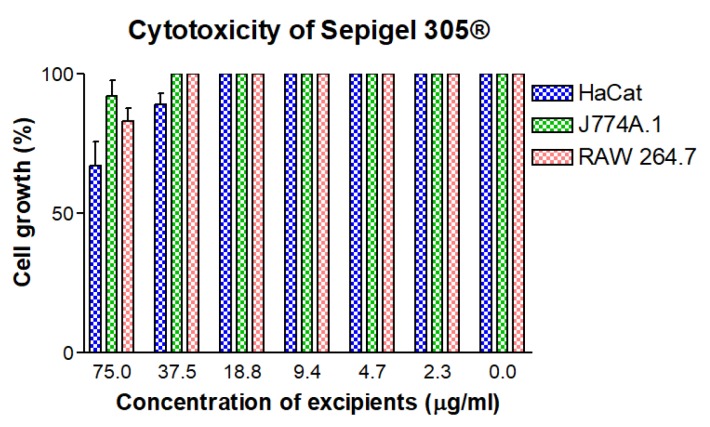
Cytotoxicity of the gelling excipients in cell lines HaCaT, RAW 264.7 and J774A.1.

**Figure 9:**
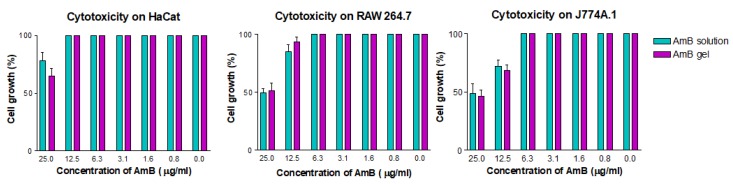
Cytotoxicity of AmB solution and AmB gel in cell lines HaCaT, RAW 264.7 and J774A.1.

As seen in Figure 8, the CC_50_ of Sepigel 305^®^ in the cell lines assayed was higher than 75.0 µg/mL. The CC_50_ of the AmB gel and solution was higher than 25.0 µg/mL (Figure 9).

### 3.9. In Vitro Leishmanicidal Activity against Promastigotes and Amastigotes

Table 3 summarizes the activity on promastigotes and amastigotes. The IC_50_ of the AmB solution and AmB gel was 0.35 ± 0.02 µg/mL and 0.56 ± 0.12 µg/mL against promastigotes, and 0.91 ± 0.07 µg/mL and 0.87 ± 0.10 µg/mLagainst amastigotes, respectively. The selectivity index (SI) values of AmB solution and AmB gel were higher than 25 for both macrophage cell lines.

## 4. Discussion

Although AmBisome^®^, an AmB liposomal formulation for intravenous administration, isvery effective for the treatment of visceral leishmaniasis, its high price limits its use in the routine treatment of CL, despite an agreement with the World Health Organization and Gilead Sciences resulting in a price reduction in developing countries [32]. A topical formulation that achieves dermal drug delivery is a goal in CL treatment, as it will allow patients to administer their own treatment, avoid the discomfort of painful injections and reduce costs.

Gels are colloidal systems used in drug delivery to the skin that may improve the distribution and stability of compounds, as well as reduce undesirable systemic effects [33]. The self-emulsifying agent Sepigel 305^®^ allows a simple and cost-effective formulation of gels for topical use, as the emulsification process does not require any heat energy [34]. In the current study, we formulated an AmB gel made with multifunctional polymer formulation Sepigel 305^®^ as a possible topical alternative in the CL therapy.

Drug release is affected by the degradation of the gel in a physiological environment and also the swelling ratio—the faster the swelling of the gel is accomplished, the greater its porosity. The degradation rate of the AmB gel was constant and completed in 10 min, indicating it was not dependent on the remaining amount of polymer. The high porosity percentage of the AmB gel was corroborated by SEM images, which revealed undulating layers uniformly arranged and spaces between the layers, resembling holes or cavities. We reported similar levels of swelling, degradation and porosity in a previously characterized meglumine antimoniate Sepigel 305^®^ [35].

Near the isoelectric point, the pH of AmB gel was 5–6, which means it is biocompatible and suitable for skin application [36]. The visual appearance of the formulation remained unchanged throughout the experiment, which was in line with the backscattering profiles used to evaluate the physical stability of the formulation over a long period of time. The superimposed graphs (Figure 3) showed variations below 10%, which is indicative of stability [37,38]. The variability in the AmB concentration at t_0_ in the analysis content could be explained because different batches of AmB gel were prepared in order to dispose of fresh formulation to perform the experiments. Moreover, the AmB content in the gel remained constant over a span of 60 days at 4 °C, indicating that the drug is chemically stable when refrigerated. By contrast, at RT the stability analysis indicated that less than 20% of the main active ingredient was detectable, and it was even lower when stored at 37 °C. This could represent a limitation for the storage and clinical manipulation, as refrigeration is not always available. These stability results differ from the marketed AmB liposome formulations (AmBisome^®^, Abelcet^®^), where the stability is guaranteed for around 24–48 h at 2–8 °C once the reconstitution has been performed. Also, Rizzo et al. (2018) found rapid AmB degradation within 2 days when analyzing its stability in different combinations of AmB solutions [39]. Other studies support the use of excipients for semisolid dosage forms to increase the stability of AmB [24,38,40].

Rheological analysis provides information about aspects of the product application. The AmB gel exhibited rheo-thinning behavior (a term used by the Rheology Society to replace pseudoplastic behavior) and high viscosity, and showed a hysteresis loop in the rheograms, indicating a probable thixotropic comportment of the gel. Thixotropic behavior is an advantage in topical formulations, favoring application, as the viscosity of the formulation varies with friction and retention on skin is enhanced [41]. Friction reduces the viscosity of thixotropic fluids, which permits a good spreadability, and the initial status of the gel is recovered once the friction stops. The high spreadability of the AmB gel, as shown in Figure 5, can be explained by the dependence of the viscosity on the shear rate and shear time. When the gel was submitted to deformation (added weights), the viscosity decreased and spreadability was enhanced.

The AmB release profile was assessed to check that the gel was well formulated and permitted the AmB liberation to monitor the kinetic behavior [42]. The data obtained demonstrated a constant release for 48 h in a Fickian diffusion process. At 48 h, the amount of AmB released through the nylon membranes was 100% for the AmB solution because there were no impediments, and it confirms the suitability of the membrane, while AmB from the gel was 66.80% liberated. It is an expectable value for a modified release formulation where the excipients play an important role in the liberation of the drug. In a comparative study, Potúcková et al. (2008) reported that drug release was increased when the excipient in the marketed formulation was replaced by Sepigel 305^®^ [43].

Permeation was studied on both damaged and non-damaged skin, as CL lesions can manifest as a nodule or a papule entirely covered with epidermis, or as an ulcer without this layer. No permeation of AmB from the gel was detected in the receptor compartment in either skin type after 24 h. In contrast, high amounts of AmB were retained in both types, with higher values in the non-damaged skin, suggesting that the stratum corneum acts as a reservoir of the drug. The reservoir effect of skin is helpful in the topical treatment of CL nodules or papules because the local depot effect potentiates the treatment duration as this kind of lesion is covered by the epidermis layer [44]. In contrast, this depot effect will not occur in ulcerated lesions. The high deposition of AmB in both kinds of skin observed in this study could indicate a long availability, as commonly required. The achieved levels of AmB retained in both kinds of skin resulted in a higher value than the IC_50_ of AmB for amastigotes, which is considered a goal for successful treatment [45].

Studies on other topical AmB formulations applied on human skin and porcine vaginal mucosa have also reported the absence of AmB in the receptor compartment and high amounts retained inside tissues [24,38]. This behavior can be explained by the high lipophilicity of AmB, which limits its ability to pass through the aqueous dermal structure. Our studies in both kinds of skin reflect that AmB is able to cross the stratum corneum to the epidermis and dermis. Our results with stripped skin (“damaged skin”) are comparable to those obtained with porcine vaginal mucosa, which does not present stratum corneum layer, and AmB can reach the dermis but is not distributed in the systemic circulation. This suggests that after topical application, part of the drug would remain in the epidermis and dermis, thus avoiding systemic side effects. As well as providing a high drug concentration in the skin, an effective topical treatment of CL should target the amastigotes of *Leishmania* parasites in the phagolysosome of infected macrophages in the papillary dermis, which is found immediately below the epidermis [46].

In contrast with these results, Jaafari et al. (2019) reported an AmB flux through mouse full-thickness skin to the receptor compartment after the application of AmB liposomes and corroborated the permeation in vivo when the same liposomes were administered in a mouse model [47]. Different studies carried out with AmB nanoemulsions formulated with surfactants for topical delivery reported a high retention of the drug in rat skin and flux to the receptor compartment [40,48,49]. The surfactants in the nanoemulsions enhance AmB permeation by destabilizing the cellular bilayer lipids in the stratum corneum, increasing deformation and enhancing the permeation [50]. It is also relevant that rat skin may lead to a greater permeation of drugs [51], as observed in studies reporting the higher permeability of rat skin compared with human skin [52,53].

The biomechanical parameters of transepidermal water loss and stratum corneum hydration were monitored for 2 h after application to evaluate skin integrity. The values obtained revealed a decrease in the TEWL immediately after AmB gel application, as well as an increased hydration of the stratum corneum due to an occlusive effect of the formulation. This was probably caused by the high lipophilicity of the drug. The TEWL and hydration of the stratum corneum basal values were recovered over 1 h after application, indicating changes in the skin are reversible. Furthermore, no discomfort after the gel application was reported by the volunteers, who did not experience any burning sensation, irritation or itching.

Sepigel 305^®^ and the AmB gel and solution did not show toxicity in the range of dilutions assayed on the three cell lines tested in the experiments. The results corroborate previous reports of safety of using Sepigel 305^®^ in topical formulations, with the exception of one case of allergic contact dermatitis [54].

No differences in the in vitro activity against *Leishmania infantum* promastigotes and amastigotes were observed between the AmB solution and the formulation, indicating an absence of a synergistic effect between the gel excipients and the AmB. 

Due to the safe usage of other AmB formulations that has been previously reported in healthy volunteers [55], and prompted by the study of López et al. (2018), who concluded that their 3% AmB cream tested in patients with uncomplicated CL needed reformulation of either the vehicle or concentration [56], we propose Sepigel 305^®^ as a suitable vehicle for 0.1% AmB for topical treatment. Allowing a good release of the AmB with high amounts of the drug retained in excised human skin, the formulation could be a potential tool for CL treatment, as only a few doses of AmB are required to kill the parasite. Further assays in an animal model should be performed to establish the antileishmanial efficacy of the AmB gel.

## 5. Conclusions

The development of a topical treatment for CL is desirable, as painful intralesional treatments are widely rejected by patients. In this context, we elaborated an AmB gel with optimal sensorial and functional properties that was shown to be stable for 60 days at 4 °C. The organoleptic characteristics of the gel are compatible with usage on skin, as it has a suitable pH, good spreadability and drug release, and did not produce irritation.

Damaged and non-damaged skin were exposed to amounts of AmB considered sufficiently high to exert local action against CL. No AmB was detected in the receptor compartment due to its high molecular weight and lipophilicity. This represents an advantage as the treatment would avoid systemic toxicity.

No cytotoxic effects were observed in the macrophage or in the keratinocyte cell lines. Although the antileishmanial activity of AmB was not affected by the gel formulation in vitro, the AmB gel should undergo in vivo testing to further evaluate its activity and efficacy in the treatment of CL.

## Figures and Tables

**Table 1:** Concentration of AmB and SD at the beginning of the experiment (t_0_ = initial time) and 60 days later (t_60_) at different storage temperatures.

AmB (µg/mL ± SD)				
	t_0_	t_60_ RT	t_60_ 37 °C	t_60_ 4 °C
AmB gel	959.90 ± 84.27	199.65 ± 27.99	70.95 ± 13.59	916.70 ± 99.78

**Table 2:** Amounts of AmB found in the permeation studies after 24 h in damaged and non-damaged skin.

Assay	AmB gel	
	Non-Damaged Skin	Damaged Skin
Permeation (µg/cm^2^) ± SD	Not detected	Not detected
Retention in skin (µg/g/cm^2^) ± SD	2484.57 ± 439.12	1230.10 ± 331.52

**Table 3:** Activity in vitro against promastigotes and amastigotes, cytotoxicity of AmB solution and AmB gel and selectivity index (SI).

Formulations (µg/mL)	IC_50_ (µg/mL ± SD)		SI		CC_50_ (µg/mL)	
	Promastigotes	Amastigotes	SI_RAW_	SI_J774A.1_	RAW 264.7	J774A.1
AmB solution	0.35 ± 0.02	0.91 ± 0.07	27.52	27.52	>25	>25
AmB gel	0.56 ± 0.12	0.87 ± 0.10	28.74	28.74	>25	>25
